# Potential benefits of *L. acidophilus* in dyslipidemic rats

**DOI:** 10.1038/s41598-021-85427-8

**Published:** 2021-03-17

**Authors:** Onrapak Reamtong, Tipparat Thiangtrongjit, Nathamon Kosoltanapiwat, Watanalai Panbangred, Pattaneeya Prangthip

**Affiliations:** 1 Department of Molecular Tropical Medicine and Genetics, Faculty of Tropical Medicine, Mahidol University, Bangkok, Thailand; 2 Department of Microbiology and Immunology, Faculty of Tropical Medicine, Mahidol University, Bangkok, Thailand; 3 Department of Biotechnology, Faculty of Science, Mahidol University, Bangkok, Thailand; 4 Department of Tropical Nutrition and Food Science, Faculty of Tropical Medicine, Mahidol University, 420/6 Ratchawithi Road, Ratchathewi, Bangkok, 10400 Thailand

**Keywords:** Dyslipidaemias, Nutrition

## Abstract

Several studies have shown that probiotics and synbiotics ameliorate dyslipidemia. However, the molecular mechanisms mediating their effects remain to be determined. Therefore, we aimed to compare the effects of a probiotic, a prebiotic, and a synbiotic in dyslipidemic Sprague–Dawley rats, and explore the mechanisms involved using a proteomic approach. The rats were allocated to five groups: a control group that was fed normal chow, and four high-fat diet-fed groups, three of which were administered a probiotic (*Lactobacillus acidophilus*), a prebiotic (inulin), or a combination of the two (a synbiotic) for 30 days. We showed that the administration of inulin, and especially *L. acidophilus*, improved the lipid profile and reduced the serum concentrations of inflammatory markers in high-fat diet-fed rats. Proteomic analysis showed changes in lipid elongation, glycerolipid metabolism, activation of antioxidants, and a reduction in the activation of the mitogen-activated protein kinase signaling pathway in the livers of rats administered *L. acidophilus*, which likely mediate its beneficial effects on inflammation and dyslipidemia by reduced the levels of 18.56% CRP, 35.71% TNF-α 25.6% LDL-C and 28.57% LDL-C/HDL-C ratio when compared to HF group. *L. acidophilus* and inulin may represent effective natural means of maintaining inflammation and dyslipidemia.

## Introduction

Overweight and obesity are major public health problems worldwide, and their prevalences have continuously increased during recent decades. In 2016, approximately 13% of adults were obese worldwide^1^, but increases in the prevalence of overweight and obesity have also occurred in children and adolescents. Overweight and obesity increase the risks of heart disease and related mortality, through the associated dyslipidemia and low high-density lipoprotein (HDL)-cholesterol concentration^2^. Dyslipidemia was reported to relate to the activation of inflammation at least by increasing the production of inflammatory cytokines such as tumor necrosis factor alpha and interleukin 6. In reverse, cytokines could disrupt lipid metabolism, which has crucial roles in the pathogenesis of atherosclerosis^3^. The consumption of energy-dense foods that contain high proportions of fats and sugars is the principal cause of overweight and obesity^4^. This can be modeled in rats by feeding them a high-fat diet (e.g., 22.59 kJ/g from fat), which induces dyslipidemia and defects in hepatic fat metabolism^5,6^.

Probiotics are live microorganisms that have health benefits for their hosts^7^. More than 500 different microbial species have been found in the human intestinal tract, and among these is *Lactobacillus acidophilus*, which is a well-known species that is commonly found in yogurt. It usually colonizes the large intestine of newborns and remains in the intestinal tract of humans until death^8^. *L. acidophilus* has been shown to be associated with greater longevity and to have a hypocholesterolemic effect^9^; for example, it lowers serum total cholesterol, special on low density lipoprotein cholesterol in pigs^10^. Prebiotics are indigestible food components that selectively stimulate the growth and activity of probiotic species in the colon, and also reduce serum total cholesterol (TC), low-density lipoprotein (LDL)-cholesterol, and triglyceride (TG) concentrations^11^. Inulin is an indigestible polysaccharide that is produced by plants, which was approved for use as a prebiotic in the United States by the Food and Drug Administration in 2018, because it improves the nutritional value of manufactured food products^12^. Specifically, inulin has been reported to reduce body weight gain, liver weight, and the serum and hepatic TC and TG concentrations in mice^13^. Furthermore, the consumption of a combination of prebiotics and probiotics, termed a synbiotic, has been reported to improve host health^14^. For example, the synbiotic mixture of *Lactobacillus plantarum* S58 and hull-less barley β-glucan has been shown to reduce lipid accumulation in high-fat diet-fed mice^15^. However, the effects of a synbiotic composed of *L. acidophilus* and inulin on lipid metabolism have not been investigated in an animal model, and the molecular mechanisms involved remain to be determined.

In the present study, we studied five groups of rats: one fed a normal diet (N), and four fed a high-fat diet (HF), three of which were also administered *L. acidophilus* (HFLac), inulin (HFIn), or both *L. acidophilus* and inulin (HFLacIn). After 30 days, blood was collected for biochemical analyses and to measure inflammatory marker concentrations. The rats were euthanized and their livers were weighed and subjected to histological and proteomic analysis. In this way we have characterized the host lipid profile associated with *L. acidophilus* and inulin consumption, and identified key proteins that are involved in the mechanisms of the effects of this synbiotic combination.

## Results

### Rat body and liver masses

**Figure 1:**
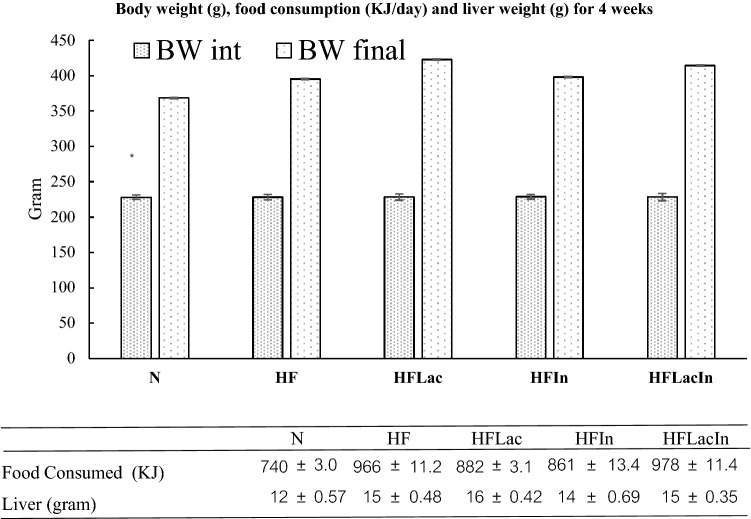
Body mass (g), food consumption (KJ/day), and liver mass (g) of the rats in each group (n = 13). (N) Rat fed a normal diet (HF) Rats fed with a high fat diet (HFLac, HFIn, HFLacIn,) Rats fed with a high fat diet and *L. acidophilus*, inulin, and *L. acidophilus* + inulin, respectively.

The body and liver masses were measured for all the groups (Fig. 1). High-fat diet-feeding for 4 weeks caused greater weight gain than the consumption of a normal diet. The HFLac and HFLacIn groups consumed 882 ± 3.1 and 978 ± 11.4 kJ/day, which represented larger energy intakes than in the other groups. However, the administration of *L. acidophilus*, prebiotic, or synbiotic for 4 weeks did not have significant effects on the body mass of high-fat diet-fed rats. The liver masses of all the HF groups were higher than that of the normal group. However, *L. acidophilus*, prebiotic, and synbiotic administration for 4 weeks did not significantly affect this parameter.

### Serum lipid profile

**Figure 2:**
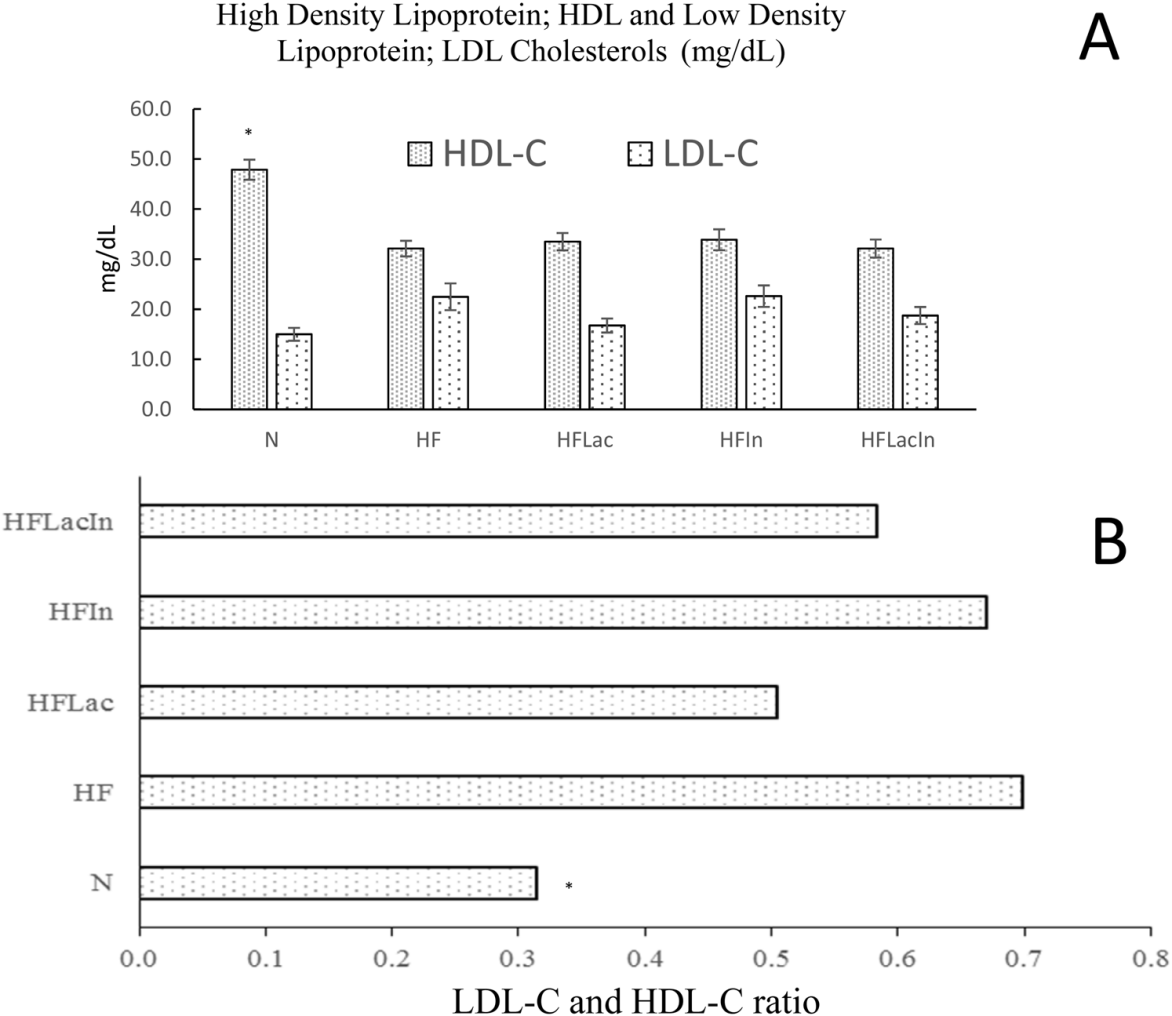
Serum HDL (mg/dL)- and LDL-cholesterol concentrations of the rats in each group (n = 13). (N) Rat fed a normal diet (HF) Rats fed with a high fat diet (HFLac, HFIn, HFLacIn) Rats fed with a high fat diet and *L. acidophilus*, inulin, and *L. acidophilus* + inulin, respectively. **(A)** Serum HDL-C and LDL-C concentrations and **(B)** the LDL-C/HDL-C ratio.

Serum lipid concentrations were measured in all the groups of rats (Fig. 2). The N group demonstrated the highest HDL-C concentration, and the concentrations were significantly lower in all the HF groups. HFLac and HFLacIn group reduced the LDL-C concentration by 25.6% and 17% respectively, and this effect was not pronounced in the HFIn group (Fig. 2A)**.** Calculation of the LDL-C/HDL-C ratio (Fig. 2B) demonstrated that the HFLac group had the most advantageous lipid profile of the HF groups by reduction of the 28.57% of LDL-C/HDL-C ratio.

### Liver and kidney-related parameters

**Figure 3:**
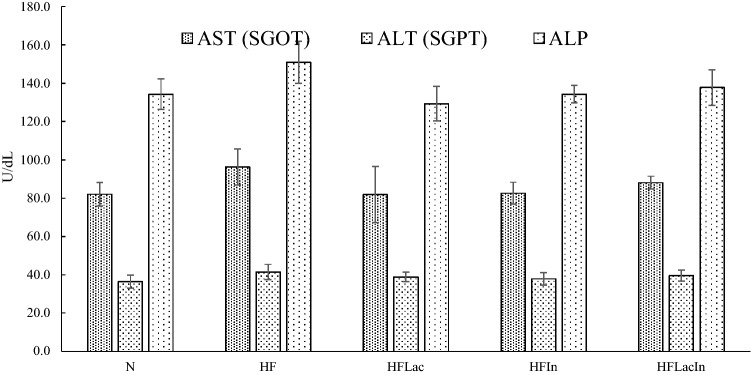
Serum liver enzyme activities (U/dL) of the rats in each group (n = 13). (N) Rat fed a normal diet (HF) Rats fed with a high fat diet (HFLac, HFIn, HFLacIn) Rats fed with a high fat diet and *L. acidophilus*, inulin, and *L. acidophilus* + inulin, respectively. *AST* aspartate transaminase, *ALT* alanine transaminase, *ALP* alkaline phosphatase.

Serum liver and kidney-related parameters were measured in all the groups (Fig. 3). The N group had the lowest AST, ALT, and ALP activities, and high-fat diet-feeding increased the activities of all these enzymes, which reflect recent liver damage, not residual function. The administration of a probiotic, prebiotic, or synbiotic combination for 4 weeks did not significantly affect the AST, ALT, or ALP activities. Kidney function was assessed by the measurement of BUN and creatinine in each group, and the values were in the ranges 11.5–13.1 mg/dl and ~ 0.2 mg/dl, respectively. No statistically significant differences were found among the groups.

### Serum inflammatory marker concentrations

**Figure 4:**
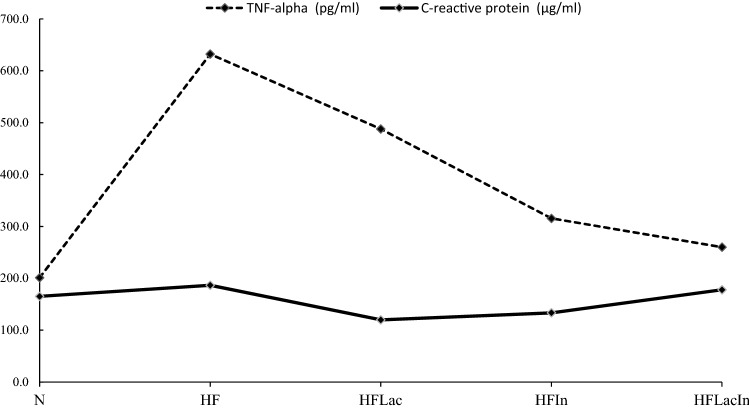
Serum concentrations of tumor necrosis factor-alpha (TNF-alpha; pg/ml) and C-reactive protein (CRP; µg/ml) of the rats in each group (n = 13). (N) Rat fed a normal diet (HF) Rats fed with a high fat diet (HFLac, HFIn, HFLacIn). Rats fed with a high fat diet and *L. acidophilus*, inulin, and *L. acidophilus* + inulin, respectively.

TNF-α is a cytokine that plays an important role in inflammation, such that a high serum concentration of TNF-α reflects systemic inflammation. We measured the serum TNF-α concentration of rats in all the groups by ELISA, and found that the serum TNF-α was higher in the HF group than in the N group (Fig. 4). However, the concentrations in the HFLac, HFIn, and HFLacIn groups were much lower than that in the HF group by 18.56–56.59%. Rats administered the synbiotic combination had the lowest TNF-α concentration among the HF groups by 56.59%. CRP is another serum marker of inflammation. The HF group exhibited a higher serum CRP concentration than the N group, and probiotic administration, but not prebiotic or synbiotic administration, reduced this by 35.71% when compared to HF group (Fig. 4).

### Hepatocyte histopathology

Histological examination of the rat livers (Supplemental Fig. 1) revealed that the hepatocytes of the HF group contained considerable more and larger fat droplets than the N group. However, no significant histological differences were found among the four HF groups.

### *Lactobacillus* identification in feces

**Figure 5:**
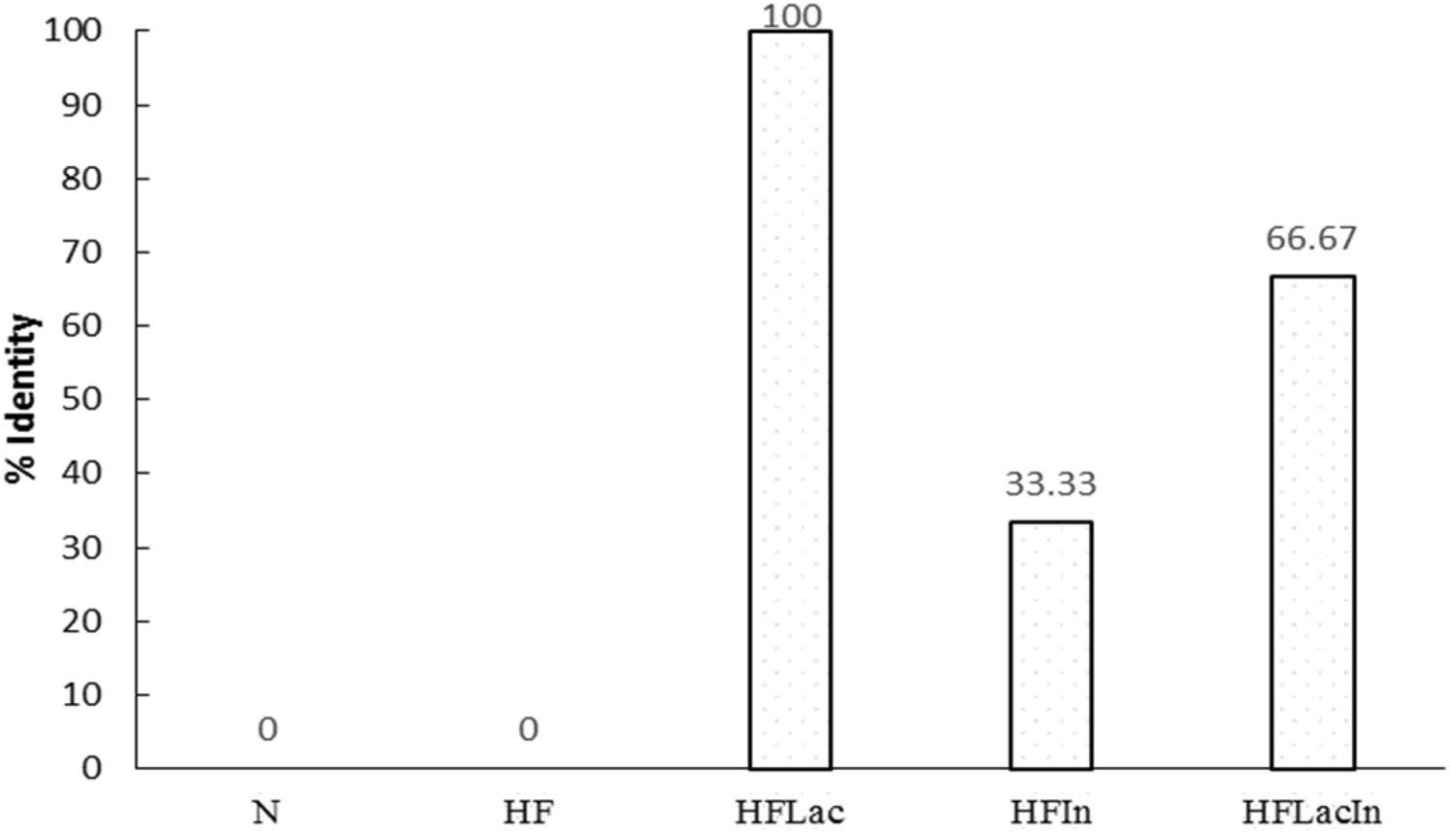
Presence of *L. acidophilus* in rat feces in each group (n = 13). (N) Rat fed a normal diet (HF) Rats fed with a high fat diet (HFLac, HFIn, HFLacIn). Rats fed with a high fat diet and *L. acidophilus*, inulin, and *L. acidophilus* + inulin, respectively. % identity values are shown.

We analyzed the feces of each rat group at the end of the study to identify the presence of *L. acidophilus* (Fig. 5). As expected, feces from the N and HF groups did not contain *L. acidophilus*. In contrast, the feces of every rat in the HFLac group contained *L. acidophilus*. Surprisingly, some of the fecal samples from rats of the HFLacIn group did not contain *L. acidophilus.* Furthermore, some of the fecal samples from the HFIn group contained *L. acidophilus.*

### Hepatic proteome

**Figure 6:**
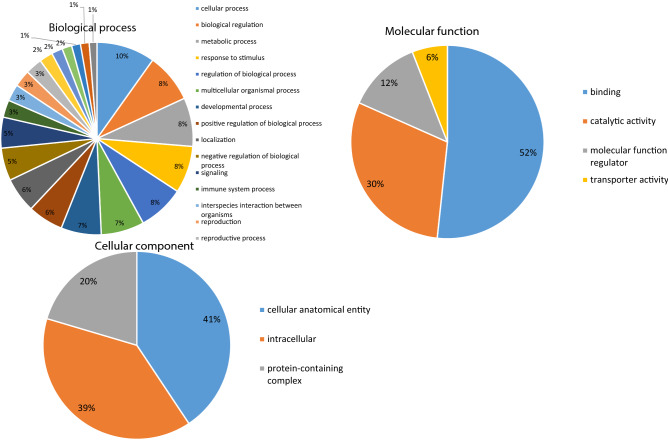
Gene ontology classification of hepatic proteins. The results of SDS-PAGE and MS analysis show the protein expression patterns of rats fed a high-fat diet (HF) and rats fed the high-fat diet and administered *L. acidophilus* (HFLac). *M* molecular marker.

The HFLac group was the group that showed the lowest LDL-C/HDL-C ratio and serum CRP concentration, and therefore we next separated the proteins in the livers of rats from the HF and HFLac groups by SDS-PAGE and stained the resulting gels using Coomassie blue (Supplemental Fig. 2). Full-length gels are presented in Supplemental Fig. 3. Next, each lane was cut into small pieces, in-gel digestion was performed, and the peptide extracts were analyzed by MS. A total of 3,944 proteins were identified in the livers. The hepatic protein profiles of the HF and HFLac groups were compared, and 534 proteins were found to be upregulated and 308 downregulated in the HFLac group. GO classification of the differentially expressed proteins demonstrated that cellular process (10%), binding (52%), and cellular anatomical entity (41%) were the major classes under the biological process, molecular function, and cellular component terms, respectively (Fig. 6). The top-20 most upregulated and downregulated proteins are presented in Tables 1 and 2, respectively.

**Table 1:** Top-20 most upregulated hepatic proteins in the HFLac group vs. the HF group.

Accession no	Protein	Fold difference	Score	M.W	pI	% Cov	No. of peptides
ENPL_RAT	Endoplasmin	5.75	566	92,713	4.72	28.6	19
RS9_RAT	40S ribosomal protein S9	4.93	154	22,578	10.66	25.3	6
A1I3_RAT	Alpha-1-inhibitor 3	4.00	755	16,3670	5.7	24.2	31
APT_RAT	Adenine phosphoribosyltransferase	3.59	73	19,533	6.17	38.3	5
HS90B_RAT	Heat-shock protein 90-beta	3.50	420	83,229	4.97	24.7	16
COX2_RAT	Cytochrome c oxidase subunit 2	3.38	91	25,925	4.6	19.4	3
A1M_RAT	Alpha-1-macroglobulin	3.00	177	16,7019	6.46	16.5	18
PHB_RAT	Prohibitin	2.92	193	29,802	5.57	39	7
TERA_RAT	Transitional endoplasmic reticulum ATPase	2.86	236	89,293	5.14	26.2	16
MUG1_RAT	Murinoglobulin-1	2.67	648	16,5221	5.68	21.3	28
HS90A_RAT	Heat-shock protein 90-alpha	2.62	210	84,762	4.93	20.2	14
CO3_RAT	Complement C3	2.50	275	18,6342	6.12	18.9	23
ACTS_RAT	Actin, alpha skeletal muscle	2.46	532	42,024	5.23	44.3	16
BUP1_RAT	Beta-ureidopropionase	2.28	117	44,014	6.47	19.6	6
GSTM4_RAT	Glutathione *S*-transferase Yb-3	2.25	305	25,664	6.84	52.3	13
HMCS2_RAT	Hydroxymethylglutaryl-CoA synthase, mitochondrial	2.21	851	56,876	8.86	46.3	22
UD16_RAT	UDP-glucuronosyltransferase 1–6	2.20	148	60,093	8.91	22.1	7
TPIS_RAT	Triosephosphate isomerase	2.17	374	26,832	6.89	60.6	11
PGAM1_RAT	Phosphoglycerate mutase 1	2.00	121	28,814	6.67	37.4	7
AL1A7_RAT	Aldehyde dehydrogenase, cytosolic 1	2.00	115	54,525	7.1	23.4	10

**Table 2:** Top-20 most downregulated hepatic proteins in the HFLac group vs. the HF group.

Accession no	Protein	Fold difference	Score	M.W	pI	% Cov	No. of peptides
UBIQ_RAT	Ubiquitin	11.15	216	8,560	6.56	78.9	7
PRDX5_RAT	Peroxiredoxin-5, mitochondrial	5.92	436	22,165	8.94	75.6	13
DYR_RAT	Dihydrofolate reductase	4.88	136	21,624	6.77	28.3	5
RS3A_RAT	40S ribosomal protein S3a	4.73	210	29,926	9.75	44.3	12
PHS_RAT	Pterin-4-alpha-carbinolamine dehydratase	3.90	150	11,992	6.28	45.2	4
RL23A_RAT	60S ribosomal protein L23a	3.63	192	17,684	10.44	25.6	5
RL17_RAT	60S ribosomal protein L17	3.44	196	21,383	10.2	29.9	5
EST5_RAT	Liver carboxylesterase B-1	3.40	121	62,455	6.25	25.3	10
EST4_RAT	Liver carboxylesterase 4	3.40	102	62,269	6.29	17.5	6
PDIA3_RAT	Protein disulfide-isomerase A3	3.33	631	56,588	5.88	39.2	17
CH10_RAT	10 kDa heat-shock protein, mitochondrial	3.28	380	10,895	8.89	84.3	11
ECHA_RAT	Trifunctional enzyme subunit alpha, mitochondrial	3.00	216	82,613	9.16	24.6	16
ARGI1_RAT	Arginase-1	2.85	202	34,951	6.76	39.9	9
RS18_RAT	40S ribosomal protein S18	2.67	267	17,708	10.99	40.1	8
RS10_RAT	40S ribosomal protein S10	2.38	239	18,904	10.15	33.3	6
RLA1_RAT	60S acidic ribosomal protein P1	2.30	63	11,491	4.28	28.9	2
COF1_RAT	Cofilin-1	2.22	107	18,521	8.22	41	5
MDHC_RAT	Malate dehydrogenase, cytoplasmic	2.21	314	36,460	6.16	33.5	11
ATPD_RAT	ATP synthase subunit delta, mitochondrial	2.21	111	17,584	5.16	18.5	3
CDC42_RAT	Cell division control protein 42 homolog	2.13	63	21,245	6.15	15.7	2

**Figure 7:**
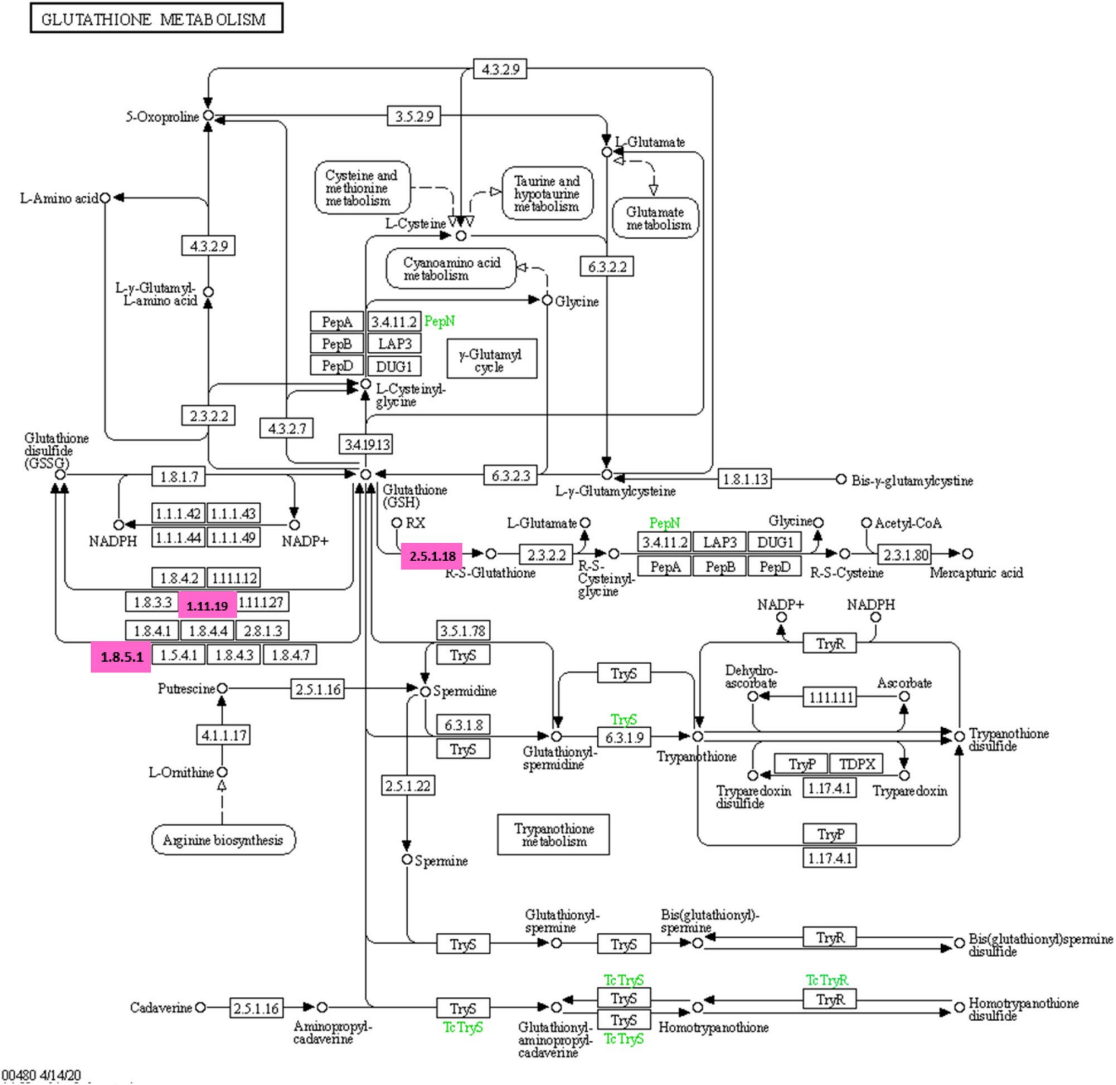
Glutathione metabolism. Pink boxes: upregulated proteins in the HFLac group.

**Figure 8:**
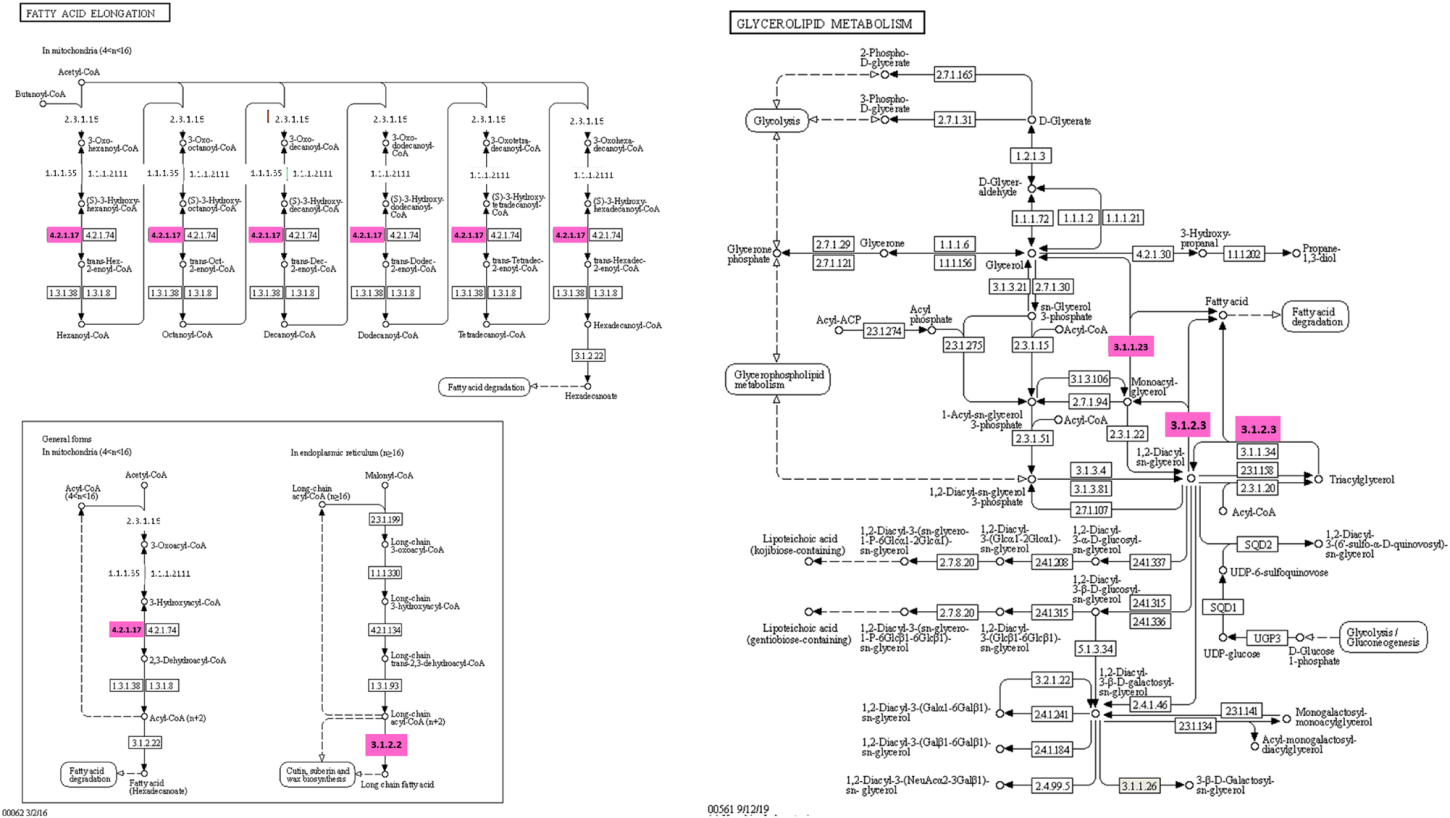
Fatty acid elongation and glycerolipid metabolism. Pink boxes: downregulated proteins in the HFLac group.

**Figure 9:**
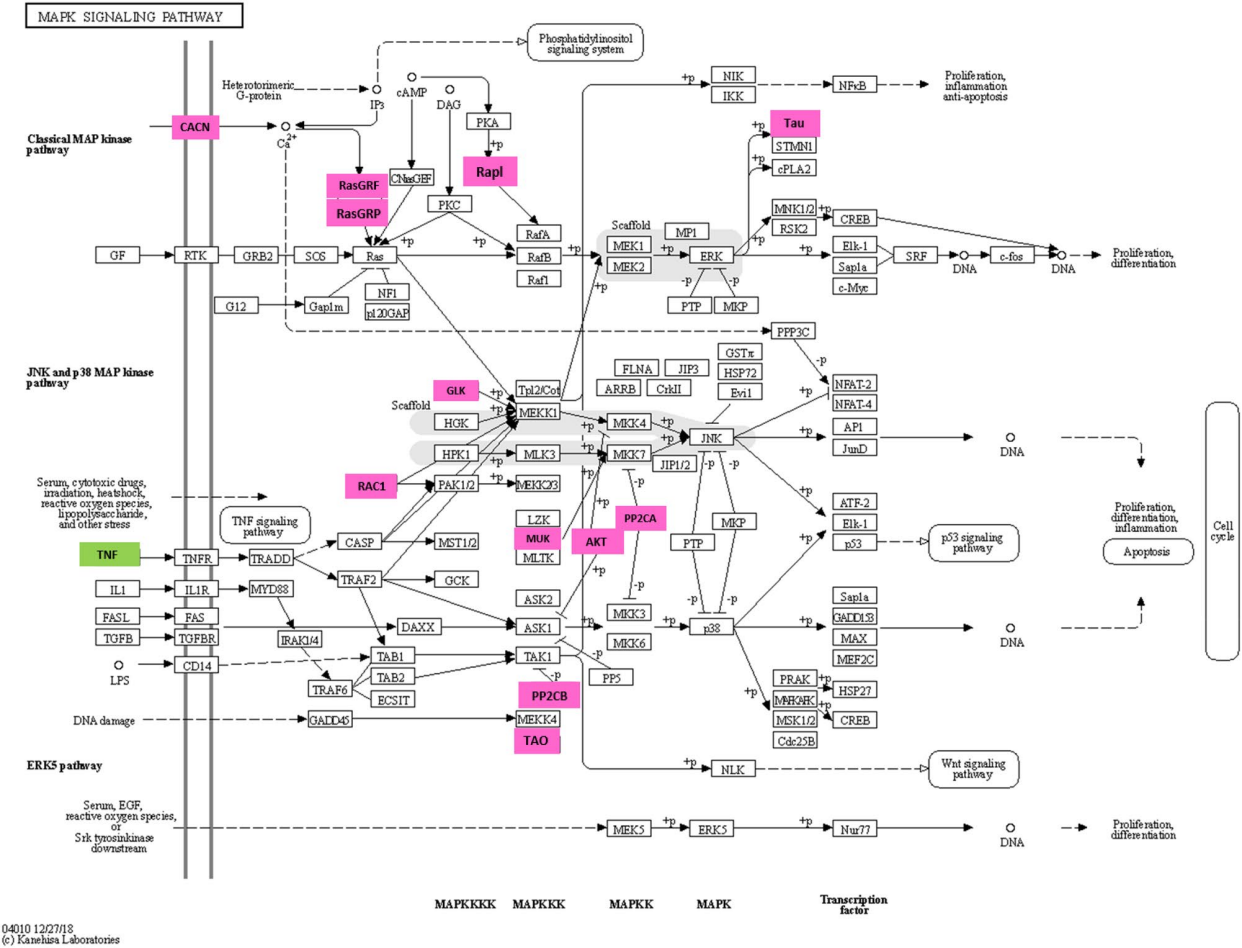
MAPK signaling pathway. Green box: tumor necrosis factor-alpha. Pink boxes: downregulated proteins in the HFLac group.

Endoplasmin, 40S ribosomal protein S9, and alpha-1-inhibitor 3 were upregulated in the HFLac group, with fold differences of 5.75, 4.93, and 4.00, respectively. Additionally, ubiquitin, peroxiredoxin-5, and mitochondrial and dihydrofolate reductase were down-regulated in the HFLac group, with fold differences of 11.15, 5.92, and 4.88, respectively. The differentially expressed proteins were then subjected to pathway analysis using Blast2Go software: this showed that glutathione metabolism was the principal upregulated pathway in the HFLac group (Fig. 7). Specifically, glutathione S-transferase Mu 2 (GSTM2_RAT), glutathione S-transferase omega-1 (GSTO1_RAT), and phospholipid hydroperoxide glutathione peroxidase (GPX41_RAT) were upregulated in the HFLac group. In contrast, fatty acid elongation and glycerolipid metabolism were downregulated in the HFLac group (Fig. 8). In the fatty acid elongation pathway, sterol O-acyltransferase 2 (SOAT2_RAT), 3-hydroxyacyl-CoA dehydrogenase type-2 (HCD2_RAT), isovaleryl-CoA dehydrogenase (IVD_RAT), and short-chain specific acyl-CoA dehydrogenase (ACADS_RAT) were downregulated in the HFLac group. With respect to glycerolipid metabolism, lipoprotein lipase (LIPL_RAT) and pancreatic lipase-related protein 1 (LIPR1_RAT) were downregulated in the HFLac group (Fig. 8). Furthermore, the mitogen-activated protein kinase (MAPK) signaling pathway was downregulated in the HFLac group (Fig. 9). This finding is consistent with the much lower serum TNF-α concentration in the HFLac group than in the HF group.

## Discussion

The feeding of a high-fat diet for 4 weeks increased the serum LDL-cholesterol concentration, reduced that of HDL-cholesterol, and abnormal hepatic fat droplets accumulation in the rats, which is consistent with dyslipidemia and fatty liver. Dyslipidemia is a well-known major risk factor for cardiovascular disease, which is a leading cause of morbidity and mortality worldwide^2^. Dietary supplementation with probiotics, prebiotics, or synbiotics has been suggested to improve cholesterol metabolism, but inconsistent findings have been made in both animal and human studies^17^. Therefore, in the present study, we assessed the effects of oral supplementation with an *L. acidophilus* probiotic, inulin, as a prebiotic, and the combination of *L. acidophilus* and inulin, as a synbiotic, in rats fed a high-fat diet.

The results demonstrate that these supplements reduce serum LDL-cholesterol in rats, and in particular, rats that were administered *Lactobacillus* (HFLac) had a superior LDL/HDL ratio than rats administered the other supplements. These findings were consistent with those of a previous study^18^, which showed that mice fed a high-fat diet and administered *L. acidophilus* (4 × 10^7^ cfu/mL) for 10 weeks had significantly lower TC and LDL-cholesterol concentrations. Furthermore, the present proteomic study demonstrated that microsomal fatty acid elongation and glycerolipid metabolism are downregulated by *L. acidophilus* intake. Microsomal fatty acid elongation is considered to be the predominant pathway for the elongation of fatty acid with ≥ 12 carbon atoms^19^. This pathway uses fatty acids derived from the diet, and its inhibition is associated with lower plasma lipid concentrations^20^. Therefore, the downregulation of fatty acid elongation in the HFLac group may contribute to the low circulating LDL-cholesterol concentration. In the glycerolipid metabolism category, lipase expression was downregulated in the HFLac group. Lipases are a subclass of the esterases that play important roles in the digestion, transport, and processing of dietary lipids^21^; therefore, inhibition of lipase activity could reduce the gastrointestinal absorption of fats, leading to their loss in the feces, rather than their full absorption^22^, and also explain the low serum LDL-cholesterol concentration. Even though our proteomic analysis changes are pointing in the same direction of our serum lipid profile and inflammation, it was our limitation that proteomics results should confirmation with molecular methods like western blotting.

Inulin is a form of soluble fiber that not digested in the human small intestine, but can be broken down by intestinal bacteria, which generates products that are beneficial for health^23^. The addition of inulin to the diet at a low concentration (0.04%) has been shown to considerably improve the growth of *L. acidophilus*^24^; therefore, we also compared the effects of *Lactobacillus* administration alone with that of this prebiotic and a combination of the two (a synbiotic) in the present study. Inulin consumption reduces circulating TC and LDL-cholesterol concentrations^25^ by increasing the deconjugation of bile acids by gut bacteria^26^. This may have been one of the mechanisms whereby inulin reduced LDL-cholesterol in the present study.

Effects of *Lactobacillus* probiotics on immune regulation have been previously described. Certain probiotic strains have positive effects on immunity because they stimulate host immune responses^27^. TNF-α and CRP regulate the immune system and protect the body from microbiological invasion by activating the inflammatory response. Increases in the concentration of either TNF-α or CRP have also been reported alongside dyslipidemia and obesity^28^. In the present study, we found that the TNF-α concentrations in the HFLac, HFIn, and HFLacIn groups were less than that in the HF group. Furthermore, the CRP concentration was lower in the HFLac group than in the HF group. Therefore, the effects of *L. acidophilus* on immunity may be mediated at least in part through reductions in the concentrations of TNF-α and CRP.

*L. acidophilus* has also been shown to increase the levels of cluster of differentiation (CD)4 + , CD4 + /CD8 + , and immunoglobulin A, while significantly reducing those of interleukin (IL)-6 and TNF-α in vitro and *in vivo*^27^. The present proteomics study showed that glutathione metabolism was an upregulated pathway in the HFLac group. Glutathione (GSH) plays important roles in the defense against oxidative stress. The cysteine residues of GSH are readily oxidized to form glutathione disulfide (GSSG) by free radicals and reactive oxygen/nitrogen species^29^. We also found the relationship of oxidation reduction and cellular biosynthetic process (Supplemental Fig. 4.). Therefore, the consumption of *L. acidophilus* may reduce oxidative stress in rats through the glutathione pathway. A reduction in oxidative stress would reduce inflammation and the secretion of the inflammatory cytokine, TNF-α, and indeed the circulating TNF-α concentrations in the HFLac, HFIn, and HFLacIn groups were much lower than those in HF rats.

Proteins of the MAPK signaling pathway were also found to be differentially expressed in the proteomic analysis. This pathway can be activated by TNF-α, and the lower serum TNF-α concentration of the HFLac group occurred alongside the downregulation of 12 proteins in the MAPK signaling pathway. The downregulation of this pathway may be associated with lower inflammation in tissues.

Some previous studies have also shown positive effects of inulin on the immune system; for example, it increases natural killer (NK) cell activity both in mice and healthy elderly people^30,31^. Additionally, another study showed that oxidative stress was ameliorated by inulin supplementation in a rat model of type 2 diabetes mellitus^32^. However, inulin showed little capacity to suppress IL-6 and TNF-α secretion in lipopolysaccharide-treated murine macrophages^33^. To our knowledge, a clear mechanism for the effects of inulin on the immune system has yet to be identified, but it might involve reductions in inflammatory mediators. In the present study, the HFLacIn group did not show superior effects to those of probiotic (HFLac group) or prebiotic (HFIn group) alone. This might be because of the administration of an inappropriate ratio of inulin and *L. acidophilus* in the HFLacIn group; this is because it has been suggested that synbiotics should induce a synergistic effect of their pro- and prebiotic components. Prebiotics should not only stimulate the growth of probiotic microbes, but also their survival^34^.

The presence of *L. acidophilus* was confirmed by RT-PCR analysis of the rat feces in the present study. The World Health Organization defines probiotics as living microorganisms that when administered in adequate amounts may provide health benefits for the host^7^. In the present study, we found that the feces of the N and HF groups did not contain *L. acidophilus*, as expected. One hundred percent of the feces of the HFLac group contained *L. acidophilus*, whereas only 66.7% of HFLacIn group did so, which might be the result of an inappropriate ratio of *L. acidophilus* and inulin administration. Furthermore, surprisingly, feces from some of the rats in the HFIn group contained *L. acidophilus*, which is consistent with the previous discovery of *L. acidophilus* in normal rat feces^35^, and suggests that the inulin was beneficial for the survival of *L. acidophilus* in the rat gastrointestinal system.

In conclusion, the probiotic, prebiotic, and synbiotic used in the present study may represent natural means of maintaining hypocholesterolemia. Our findings suggest that the mechanisms of the beneficial effects of *L. acidophilus* in rats involve reductions in lipid elongation and glycerolipid metabolism, activation of antioxidant mechanisms, and less inflammation because of downregulation of the a mitogen-activated protein kinase (MAPK) signaling pathway. A fascinating future research should be investigated more detail dose-dependent manner.

## Methods

### Preparation of probiotics and diets

*L. acidophilus* was obtained in freeze-dried form (Natural Organic laboratories, Inc.,: Amityville, NY, USA) at a concentration of 4 × 10^7^ colony-forming units per gram (CFU/g) and stored in opaque packaging at − 20 °C. Inulin was obtained from COSUCRA: Warcoing Belgium. The rat diets were prepared according to the method of Udomkasemseb and colleagues^6^. The proportions of casein, corn starch, DL-methionine, mineral and vitamin mixtures, choline bitartrate, and corn oil were the same in each diet, and those of sucrose, cellulose, lard, and inulin were varied. The sucrose and lard contents of the high-fat diets were 33% and 17%, respectively. Inulin was included in the high-fat diet by replacing cellulose, to 1% of the total. The energy content of the normal diet was 1610 kJ/g, comprising carbohydrate (C) 67.53%, protein (P) 20.78%, and fat (F) 11.69% per unit mass. The energy content of the high-fat diets was 1966 kJ/g, comprising C 40.85%, P 17.02%, and F 42.14%. Fresh diet was provided for the rats at 10:00 every day. Probiotic was orally administered with 1.5 ml of *L. acidophilus* to the appropriate groups by reconstituted 1 g of probiotics in 1.5 ml of 0.9% sodium chloride solution (4 × 10^7^ cfu/ml), and the other groups were administered 1.5 ml of sodium chloride solution per rat.

### Animals and experimental design

The animal study protocol was approved by the Faculty of Tropical Medicine Animal Care and Use Committee, Mahidol University with the approval number FTM-ACUC 023/2561 and in compliance with the ARRIVE guidelines. All methods were carried out in accordance with relevant guidelines and regulations. The sample size was calculated using the formula of n = [(*Z*_1-α/2_ + *Z*_1-β_)^2^ (*σ*_1_^2^_+_ ([*σ*_2_^2^/r]) ]/*δ*^2^ n = sample size, *σ* = standard deviation, r = n_2_/n_1_, *δ* = difference between two group of means, power (1 − β ) = 0.9 and type I error (α) = 0.01. Ninety-one 5-week-old male Sprague–Dawley rats, weighing approximately 100–120 g, were obtained from Nomura Siam International (Bangkok, Thailand). Health certificate of rats was issued by M Clea Bioresources Co., Ltd (Bangkok, Thailand). The rats were acclimated to their new surroundings for 7 days with 2 or 3 rats housed per cage, during which period standard rat chow and water were available ad libitum. After that, the rats were simple randomly allocated by chance to five groups (n = 13 each): the rats fed with a normal diet (N), rats fed with a high fat diet (HF), rats fed with a high fat diet and inulin (HFIn), rats fed with a high fat diet and *L. acidophilus* (HFLac) and rats fed with a high fat diet, *L. acidophilus* and inulin (HFLacIn), which were fed ad libitum for 30 days. The groups being compared, are including N and HF groups. Inclusion and exclusion criteria were not set during the experiment. To minimize potential confounders between the groups, the cage of each treatments were separately located in sequence. The food consumption and body mass of the rats was measured weekly. Feces were collected on day 30 for *Lactobacillus* identification. At the end of the experimental period, the rats were fasted overnight for around 12–16 h and were then euthanized using carbon dioxide, according to the procedure of the Laboratory Animal Science Unit of the Faculty of Tropical Medicine. Blood was collected subsequently from the vena cava for biochemical analysis and the measurement of inflammatory marker concentrations. Livers were collected, weighed, and prepared for histological and proteomic analyses.

### Biochemical analyses and measurement of inflammatory marker concentrations

Three milliliters of whole blood were collected from each rat into potassium-EDTA tubes for immediate measurement of the white blood cell count using automated Cobas 6800/8800 systems (Roche Group, Switzerland). Additional blood was collected into plain tubes and centrifuged at 3000×*g* and 5 °C for 15 min. The obtained serum was stored at − 80 °C until use. One milliliter of serum per rat was used for lipid profiling (TC, TG, LDL-cholesterol, and HDL-C), the assessment of renal function (blood urea nitrogen (BUN) and creatinine concentrations), and liver enzyme activity measurement (alanine aminotransaminase (ALT), aspartate transaminase (AST), and alkaline phosphatase (ASP)) using the Cobas 6800/8800 systems. Two hundred microliters of serum were used to measure the concentrations of tumor necrosis factor-alpha (TNF-α) (Abcam: Cambridge, UK) and c-reactive protein (CRP) (Sigma-Aldrich, Inc.: St. Louis, MO, USA) using rat elisas, according to the manufacturers’ instructions.

### Histological examination

The left lateral lobes of the rat livers were sectioned transversely and fixed in 10% buffered formalin for 48 h. The livers were then embedded in paraffin, sectioned at 5.0 μm thickness, and stained with hematoxylin and eosin, prior to examination under a light microscope (Zeiss Imager.M2, Oberkochen, Germany).

### Proteomic analysis

Rat liver samples from the same group were pooled, frozen in liquid nitrogen, and ground using a mortar. Lysis buffer containing 1% SDS (Merck, Germany), 1% triton X-100 (Merck) and 1% nacl (Merck) was then added and lysates were prepared using an ultrasonicator (Sonics & Materials, USA). The lysates were then centrifuged at 12,000×*g* for 5 min at 4 °C, the supernatants were collected, and their protein concentrations were measured using a Quick Start Bradford Protein Assay (Bio-Rad, USA). Lysate aliquots containing 30 µg of protein were separated by 12% SDS-PAGE (Bio-Rad) and the gels generated were stained using Coomassie G-250 solution (Bio-Rad). Each lane was then cut into 13 pieces and the proteins within subjected to tryptic in-gel digestion^16^. The trypsin-digested fractions were separated using an Ultimate 3000 Nano-LC system (Dionex; Surrey, UK) at a flow rate of 300 nl/min. Mobile phase A was 2% (v/v) acetonitrile and 0.1% (v/v) formic acid in HPLC-grade water and mobile phase B was 0.1% (v/v) formic acid in HPLC-grade acetonitrile. The peptides were then analyzed using a micrOTOF QII mass spectrometer (MS) (Bruker; Bremen, Germany) and Hystar software. The MS and MS/MS spectra were acquired in *m/z* ranges of 400–2000 and 50–1500, respectively.

### Proteomic data analysis

The raw MS data were converted into a Mascot generic file (.mgf) using data analysis software (Mascot v.2.3.0; Matrix Science, London, UK), and this was used for protein identification using the Swissprot database. The parameters were set to *Mus musculus* (taxonomy), trypsin (enzyme), one (missed cleavage site), 1.2 Da (mass tolerance for the precursor), and Swissport 0.6 Da (mass tolerance for fragment ions). The oxidation of methionine and carbamidomethylation of cysteine residues were allowed as modifications. Only proteins with 95% confidence were reported. The exponentially modified protein abundance index (empai) was used for protein semi-quantification. Protein profiles were classified according to gene ontology (GO) using Blast2Go software and pathway analysis was performed using the Kyoto Encyclopedia of Genes and Genomes (KEGG) database ^36,37^.

### *Lactobacillus* identification

Fresh fecal samples from three rats per group were collected and stored at − 80 °C until analyzed. DNA was extracted using a qiaamp DNA stool mini kit (Qiagen, USA) from 100 mg feces and its purity was assessed using a nanodrop 2000 spectrophotometer (Thermo Fisher Scientific, USA). DNA from *L. acidophilus* was identified by real-time PCR amplification using itaq Universal SYBR Green Supermix (Bio-Rad). The reaction mixtures comprised 12 μl of SYBR Green Supermix, 2 μl each of the forward and reverse primer (10 μm), and 6.0 μl nuclease-free water. Two microliters of DNA samples were then added to each reaction mixture and amplified by thermal cycler (C1000 Touch, Applied Biosystems, USA) using an initial activation step (95 °C for 5 min), followed by 40 cycles of denaturation (95 °C for 15 s), annealing (57 °C for 15 s), and extension (60 °C for 15 s); and a final step of 65 °C for 5 s and 95 °C for 5 s. The forward and reverse primer sequences (5′–3′) for *L. Acidophilus* detection were TGCAAAGTGGTAGCGTAAGC and CCTTTCCCTCACGGTACTG, respectively.

### Statistical analysis

Body mass, biochemical parameters, inflammatory marker concentrations, and fecal *Lactobacillus* data are presented as means ± standard deviations (SD) after assessing for normality with Shapiro–Wilk test. Data were analyzed using one-way analysis of variance (ANOVA) and differences between groups were further analyzed using Duncan’s multiple range tests. Statistical significance was accepted when *P* < 0.05. Statistical analysis was performed using SPSS Statistics software Version 18 (IBM Inc., Armonk, NY, USA).

## Supplementary Information

Supplementary Figures.

## Data Availability

The data and materials supporting this article are available in faculty of tropical medicine, Mahidol University, Thailand.
