# Supplementary material for: Potential benefits of L. acidophilus in dyslipidemic rats
Source: Sci Rep. 2021 Mar 17;11:6115. doi: 10.1038/s41598-021-85427-8 (PMC7969609; doi:10.1038/s41598-021-85427-8)
Supplement: Supplementary file 1 — Supplementary Figures. [file 41598_2021_85427_MOESM1_ESM.pdf]

SUPPLEMENTAL INFORMATION FILE.

Potential Benefits of *L. acidophilus* in Dyslipidemic Rats.

Onrapak Reamtong, Tipparat Thiangtrongjit, Nathamon Kosoltanapiwat Watanalai Panbangred,

and Pattaneeya Prangthip

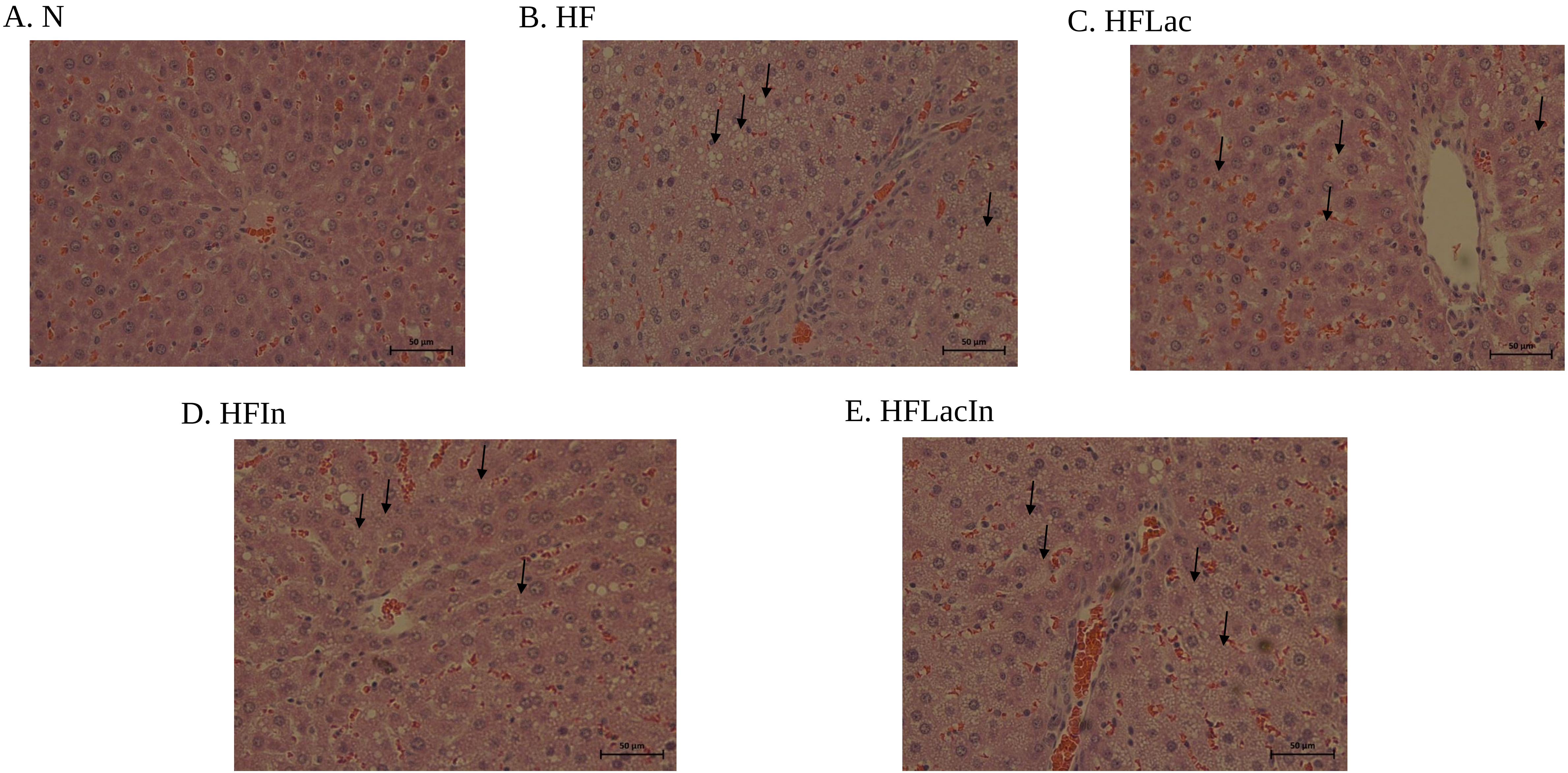

Supplemental Fig 1. Hepatic histology of rats after 30 days of treatment

Potential Benefits of *L. acidophilus* in Dyslipidemic Rats. Onrapak Reamtong, Tipparat Thiangtrongjit, Nathamon Kosoltanapiwat Watanalai Panbangred, and Pattaneeya Prangthip

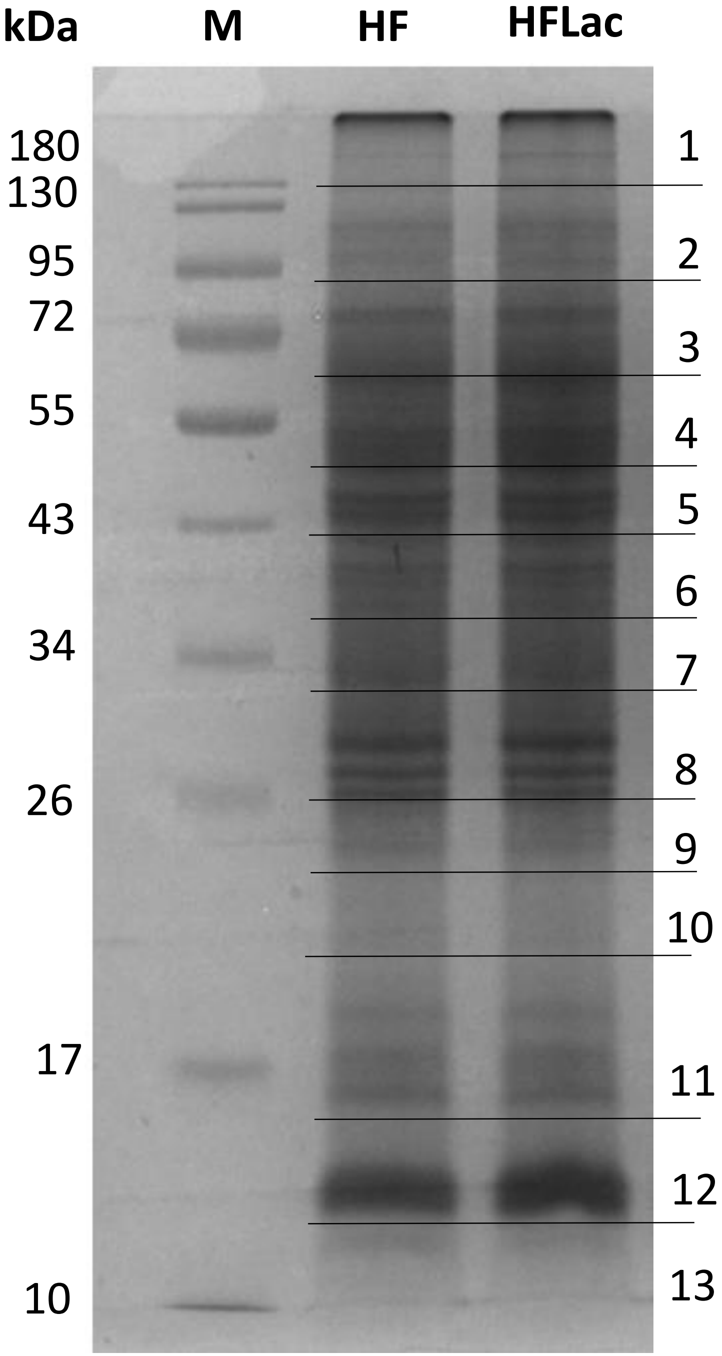

Supplemental Fig 2. Hepatic protein profile analysis. SDS-PAGE shows protein patterns of rats fed with a high fat diet (HF) and rats fed with a high fat diet and *L. acidophilus* (HFLac). M is molecular size marker.

Potential Benefits of *L. acidophilus* in Dyslipidemic Rats. Onrapak Reamtong, Tipparat Thiangtrongjit, Nathamon Kosoltanapiwat Watanalai Panbangred, and Pattaneeya Prangthip

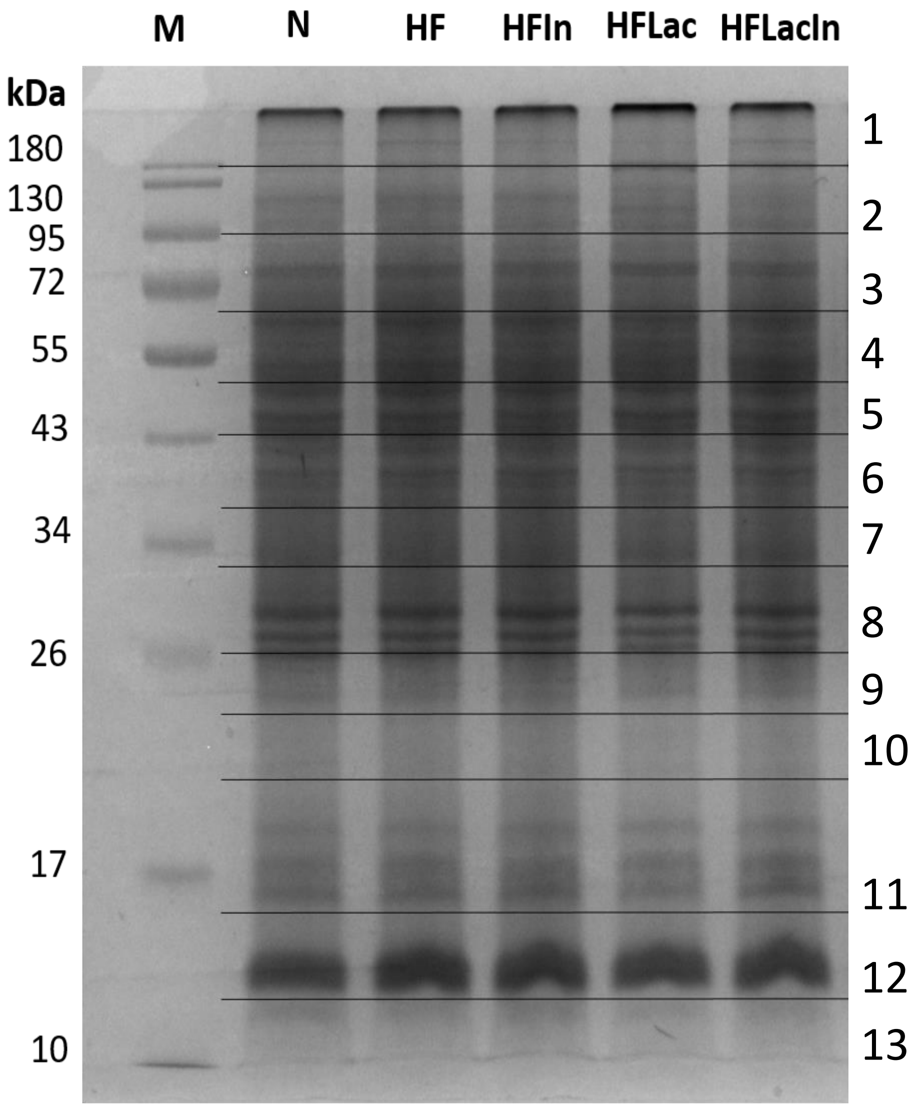

Supplemental Fig 3. Full-length gels Hepatic protein profile analysis by SDS-PAGE. The gel shows protein patterns of rats fed with a normal diet (N), rats fed with a high fat diet (HF), rats fed with a high fat diet and inulin (HFIn), rats fed with a high fat diet and *L. acidophilus* (HFLac) and rats fed with a high fat diet, *L. acidophilus* and inulin (HFLacIn). M is molecular size marker.

Potential Benefits of *L. acidophilus* in Dyslipidemic Rats. Onrapak Reamtong, Tipparat Thiangtrongjit, Nathamon Kosoltanapiwat Watanalai Panbangred, and Pattaneeya Prangthip

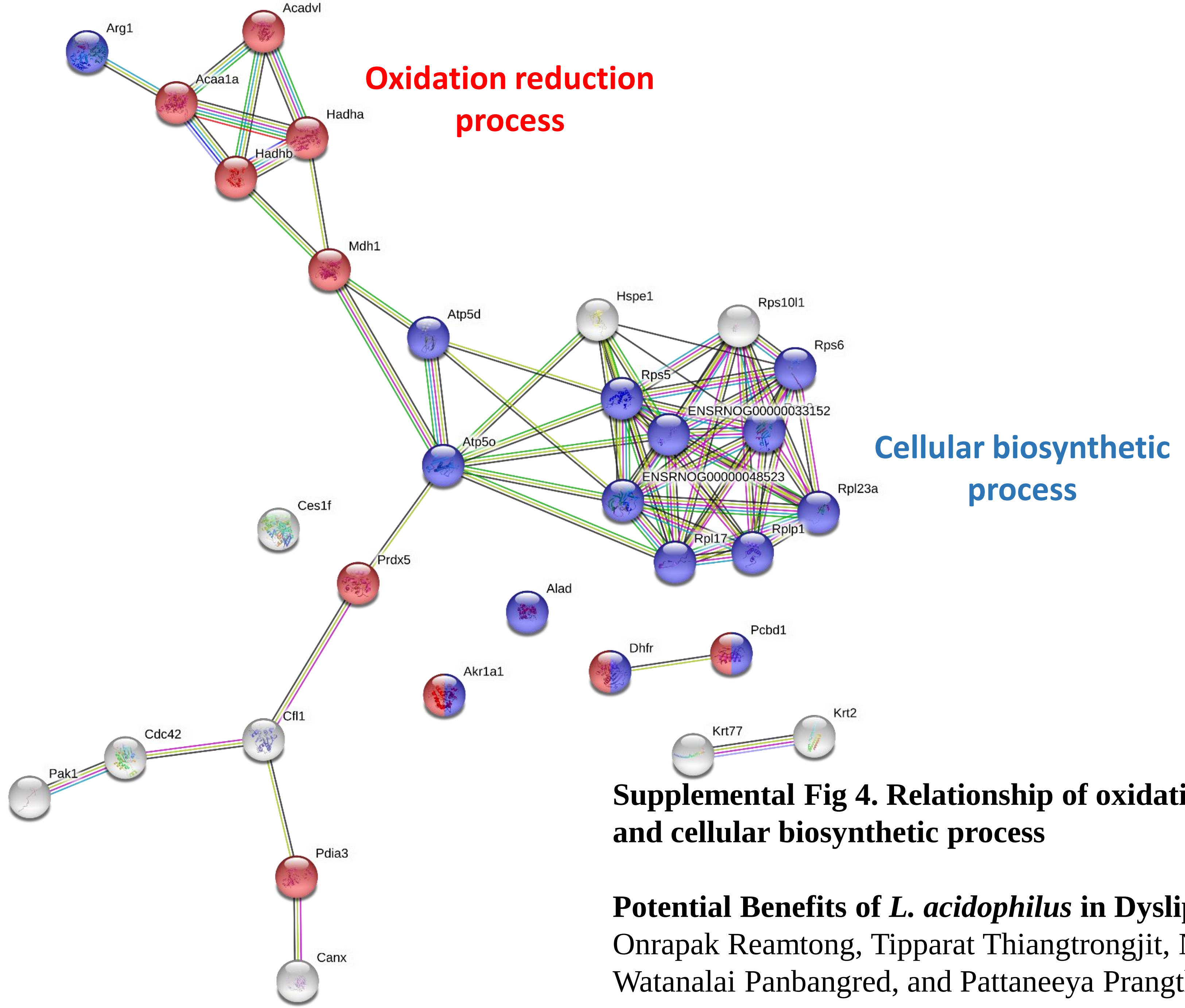

Supplemental Fig 4. Relationship of oxidation reduction and cellular biosynthetic process

Potential Benefits of *L. acidophilus* in Dyslipidemic Rats.

Onrapak Reamtong, Tipparat Thiangtrongjit, Nathamon Kosoltanapiwat Watanalai Panbangred, and Pattaneeya Prangthip
